# ﻿New faunistic records of the families Bolitophilidae and Keroplatidae (Diptera, Sciaroidea) from Morocco

**DOI:** 10.3897/zookeys.1128.85536

**Published:** 2022-11-07

**Authors:** Ouafaa Driauach, Peter J. Chandler, Mohamed Amin El Mouden, Boutaïna Belqat

**Affiliations:** 1 LESCB URL/CNRST N°18, FS, Abdelmalek Essaadi University, Tetouan, Morocco; 2 Biotechnology, Environmental Technology and Valorization of Bio-Resources Team, FSTH, Abdelmalek Essaadi University, Tetouan, Morocco; 3 606B Berryfield Lane, Melksham, Wiltshire SN12 6EL, UK

**Keywords:** Biodiversity, fungus gnats, new records

## Abstract

The family Bolitophilidae is recorded for the first time from Morocco with one species Bolitophila (Bolitophila) saundersii (Curtis, 1836). Ten new species are added to the Moroccan fauna of Keroplatidae, known until now by only two species, raising the number of species currently known in Morocco to 12.

## ﻿Introduction

The Keroplatidae are among the larger and most conspicuous fungus gnats. The family has a worldwide distribution, with about 1000 species belonging to 90 genera (Pape et al. 2011).

Keroplatids are commonly found in moist forests, but also in other ecosystems, where they are often associated with fungi, rotten wood, and similar substrates. Their larvae live in webs and either feed on fungal spores or are predaceous on small invertebrates caught in their webs. The webs may include droplets of fluid containing oxalic acid which immobilises prey. Adults are often found in dark, humid places, including caves. Keroplatids can be collected by sweeping in low vegetation, under hanging rocks, tree trunks, and along stream banks. They are also frequently caught in Malaise traps (Evenhuis 2006).

The Bolitophilidae is a small family of fungus gnats, currently comprising 61 extant species in a single genus, *Bolitophila* Meigen, 1818, which has two subgenera, *Bolitophila* s. str. and *Cliopisa* Enderlein, 1936 (Bechev and Chandler 2011). This is a principally Holarctic family, with only one species previously recorded from North Africa, in Algeria (Burghele-Balacesco 1966). Their larvae develop internally in soft fungi.

The fungus gnats of the families Bolitophilidae and Keroplatidae of Morocco are practically unstudied. This paper is the first contribution to specifically treat both families from Morocco. The family Bolitophilidae is recorded for the first time from Morocco by one species Bolitophila (Bolitophila) saundersii (Curtis, 1836). The Moroccan fauna of keroplatids was represented by only two species, *Macrocerafasciata* Meigen, 1804 (Becker and Stein 1913; Chandler and Ribeiro 1995; Evenhuis 2006; Kettani et al. 2022) and *Keroplatusreaumurii* (Dufour, 1839) (Matile 1986; Chandler et al. 2006; Evenhuis 2006). The new findings increase the number of Moroccan keroplatids to 12. Of the 10 species recorded from Morocco for the first time, six are new to North Africa.

## ﻿Material and methods

A total of 25 specimens of Keroplatidae were collected by sweeping. Between 2013 and 2022, 15 sites were sampled in mountainous areas, such as the Rif and the High Atlas.

Most of the material was collected by B. Belqat and O. Driauach, and Dr M. Ebejer provided additional material was that he had collected. All the material is preserved in 70% ethanol and was identified by P. Chandler. A list of sampling sites, with coordinates and altitudes, is given in Table 1. General and North African distributions of the species are separately given.

**Table 1.** Sampling sites (in alphabetical order) harbouring the species collected in Morocco, in the present study, with localities, geographical coordinates and elevations.

Station	Locality	Elevation (m)	Geographical coordinates
**RIF**			
Aïn Ras El Ma	Majjou	856	35°06.873'N, 5°11.388'W
Bab Rouida	Parc National Talassemtane	1512	35°06.881'N, 5°08.270'W
Daya Amsemlil	Jbel Bouhachem	1059	35°15.596'N, 5°25.917'W
Douar Belwazen	Belwazen	176	35°40.368'N, 5°25.116'W
Lower Loukkos saltmarsh	5 km E of Larache	2	35°12.274'N, 6°08.222'W
Halouma Kitane	Kitane, Tétouan	140	35°31.912'N, 5°19.861'W
Jbel Zemzem	Jbel Zemzem	216	35°45.457'N, 5°22.189'W
Marabout El Khaloua	Dar Khennouss	788	35°29.039'N, 5°20.678'W
Maison forestière	Parc National Talassemtane	1674	35°08.076'N, 5°08.262'W
Oued Aârate	Dardara	269	35°07.381'N, 5°17.456'W
Oued Majjou	Majjou Village	799	35°06.186'N, 5°10.935'W
Oued Tizga	Amsa	516	35°26.237'N, 5°13.694'W
Oued Sahel	Ben Karrich, Tétouan	40	35°29.238'N, 5°26.352'W
Oued Sidi Yahya Aârab	Sidi Yahya Aârab	62	35°17.545'N, 4°53.503'W
**High Atlas**			
Douar Akhlij Tnine Ourika	Ourika, Marrakech	870	31°22.385'N, 7°46.608'W

## ﻿List of species

### ﻿Family Bolitophilidae

#### Genus *Bolitophila* Meigen, 1818

***Bolitophilasaundersii* (Curtis, 1836)**

**Material examined**: Daya Amsemlil, 1 ♂ 1 ♀, 26 Mar. 2016.

**General distribution**: Palaearctic.

**North African distribution**: Algeria. **New record for Morocco**.

### ﻿Family Keroplatidae

**Subfamily Macrocerinae**

#### Genus *Macrocera* Meigen, 1804

***Macrocerafasciata* Meigen, 1804**

**Material examined**: Aïn Ras El Ma, 1 ♂, 1 ♀, 27 Mar. 2013.

**General distribution**: Palaearctic.

**North African distribution**: Morocco.

***Macroceranigricoxa* Schiner, 1863**

**Material examined**: Oued Aârate, 2 ♂♂, 26 Mar. 2014; Oued Sahel, 1 ♂, 5 Apr. 2014.

**General distribution**: Palaearctic.

**North African distribution: first record for Morocco and North Africa**.

***Macroceraphalerata* Meigen, 1818**

**Material examined**: Daya Amsemlil, 1 ♂, 23 Apr. 2016.

**General distribution**: Palaearctic.

**North African distribution**: Tunisia. **First record for Morocco**.

***Macrocerapusilla* Meigen, 1830**

**Material examined**: Lower Loukkos saltmarsh, 4 ♀♀, 10 May 2012, Ebejer Leg.

**General distribution**: Palaearctic.

**North African distribution**: Algeria and Tunisia. **First record for Morocco**.

##### Subfamily Keroplatinae Rondani, 1856

**Tribe Keroplatini Rondani, 1856**

#### Genus *Keroplatus* Bosc, 1792

***Keroplatusreaumurii* (Dufour, 1839)**

**Material examined**: Douar Belwazen, 1 ♂, 2 Feb. 2022.

**General distribution**: Palaearctic.

**North African distribution**: Morocco.

##### Tribe Orfeliini Malloch, 1917

#### Genus *Antlemon* Loew, 1871

***Antlemonhalidayi* Loew, 1871**

**Material examined**: Daya Amsemlil, 1 ♂, 23 Apr. 2016; Halouma Kitane, 1 ♂, 1 Jan. 2015; Oued Majjou, 3 ♂♂, 9 Apr. 2013.

**General distribution**: Palaearctic.

**North African distribution**: Algeria and Tunisia. **First record for Morocco**.

***Antlemonservulum* (Walker, 1836)**

**Material examined**: Bab Rouida, 1 ♂, 17 Jun. 2014.

**General distribution**: Palaearctic.

**North African distribution: first record for Morocco and North Africa**.

#### Genus *Macrorrhyncha* Winnertz, 1846

***Macrorrhynchagallica* Chandler & Blasco-Zumeta, 2001**

**Material examined**: Maison forestière, 1 ♂, 7–17 Jun. 2014, Malaise trap.

**General distribution**: Europe.

**North African distribution: first record for Morocco and North Africa**.

#### Genus *Neoplatyura* Malloch, 1928

***Neoplatyurabiumbrata* (Edwards, 1913)**

**Material examined**: Douar Arikji Ltnin Ourika, 1 ♂, 28 Apr. 2015; Jbel Zemzem, 1 ♂, 17 Apr. 2014.

**General distribution**: Europe.

**North African distribution: first record for Morocco and North Africa**.

***Neoplatyuranigricauda* (Strobl, 1893)**

**Material examined**: Marabout El Khaloua, 1 ♂, 3 Jun. 2018.

**General distribution**: Palaearctic.

**North African distribution**: Tunisia. **First record for Morocco**.

#### Genus *Orfelia* Costa, 1857

***Orfeliapersimilis* Caspers, 1991**

**Material examined**: Bouhachem, 1 ♂, 14 Jul. 2013; Oued Tizga, 1 ♂, 25 Jun. 2014; Oued Sidi Yahya Aârab, 1 ♂, 25 Apr. 2015.

**General distribution**: Europe.

**North African distribution: first record for Morocco and North Africa**.

#### Genus *Pyratula* Edwards, 1929

***Pyratulaebroensis* Chandler & Blasco-Zumeta, 2001**

**Material examined**: Maison forestière, 1 ♂, 7 Jun. 2014.

**General distribution**: Europe.

**North African distribution: first record for Morocco and North Africa**.
